# Do you work while sick?

**DOI:** 10.31744/einstein_journal/2021CE6150

**Published:** 2021-03-18

**Authors:** Alexsandro Tartaglia, Marcos Antonio Almeida Matos, Mary Gomes Silva

**Affiliations:** 1 Escola Bahiana de Medicina e Saúde Pública SalvadorBA Brazil Escola Bahiana de Medicina e Saúde Pública, Salvador, BA, Brazil.

Dear Editor,

Health professionals can contribute for transmission of influenza-like illness to susceptible patients while providing care.^(^^1^^)^ One asymptomatic professional may transmit pathogens directly to others and contaminate shared surfaces with touch, cough, and sneeze if not wearing a facemask.^(^^1^^,^^2^^)^ Little attention has been given in an attempt to understand and prevent pathogens transmission from health professionals to their patients.^(^^2^^)^

A study published in 2017 in the American Journal of Infection Control suggested that health care workers should be at home when they are sick. However, four out of 10 caregivers in the USA work while they are sick.^(^^3^^)^ The study was conducted using a national online survey and collected 1,914 responses from health professionals who had the influenza between 2014 and 2015. Interviewers reported influenza like symptoms, defined as a combination of fever and cough or sore throat, and they listed factors that led them not to be off sick, according to the following main reason:

– Of 1,914 health workers participating in the survey, 414 reported influenza like symptoms. Of these, 183 (41.4%) reported work for mean period of 3 days while experience the symptoms.
– Health professionals who developed their activities at hospitals had a higher frequency of work while with influenza symptoms (49.3%) compared with other professionals. Among them, those who were most likely to work with the influenza were pharmacists (67.2%) and physicians (63.2%). Nurses presented a frequency of 37.9% and among other health workers the rate was 32.1%.
– Most common reasons for health professional not be off sick included the feeling that they still able to perform their job activities, not feeling bad enough to miss work and stay at home, the feeling that they were not vehicle transmission, the feeling that they should be at their work due to professional obligation to their coworkers, and the difficult to find someone to cover for them.

In a previous study, there was the evidence that, in the hospital environment, inpatients exposed to at least one sick health professional had five times more changes to acquire influenza in the hospital than hospitalized patients without such exposition.^(^^4^^)^

Health and well-being of patients are at risk when contaminated health professionals choose to work while sick. Strategies must be adopted by health institutions, including the updating of policies for sick leave and also the provision of education for health professionals to help them to conduct healthy choices not only for them, but also to their coworkers and patients.^(^^3^^)^

In the pandemic caused by the novel coronavirus 2019 (COVID-19), which has caught the world by surprise and caused an impact on life of individuals across the globe, this is important to drawn the attention to the effects that this outbreak had achieved and to the speed at which the disease had spread. Special attention is need concerning the transmission of respiratory diseases among health professionals and patients.^(^^5^^)^ The transmission of pathogens by health professionals represents a great risk to the public health.^(^^1^^,^^3^^)^ Why not turn our attention to the issue? In fact, given the current situation, to consider this risk is a priority.

We finish this short letter, but the debate, which is complex, must continue. We believe that the issues addressed here are important points to be understood, particularly in such an unusual year as 2020, in order to guarantee a safe health care. All health workers should stay home if they are sick!
