# Supplementary material for: Do you work while sick?
Source: Einstein (Sao Paulo). 2021 Mar 18;19:eCE6150. doi: 10.31744/einstein_journal/2021CE6150 (PMC7971152; doi:10.31744/einstein_journal/2021CE6150)
Supplement: Translation

# Translation: Você trabalha doente?

**Authors:** Alexsandro Tartaglia, Marcos Antonio Almeida Matos, Mary Gomes Silva

**Affiliations:** 1 SalvadorBA Brasil Escola Bahiana de Medicina e Saúde Pública, Salvador, BA, Brasil.

Caro Editor,

Profissionais de saúde doentes podem contribuir para a transmissão de doenças semelhantes à gripe a pacientes suscetíveis enquanto prestam assistência.^(^^1^^)^ Um profissional sintomático pode transmitir patógenos diretamente para outros e contaminar superfícies compartilhadas por meio do toque, tosse e espirros sem a devida proteção.^(^^1^^,^^2^^)^ Pouca atenção tem sido direcionada à compreensão e à prevenção da transmissão de patógenos de profissionais de saúde para pacientes.^(^^2^^)^

Estudo publicado em 2017 no *American Journal of Infection Control* sugere que os profissionais de saúde devem ficar em casa quando doentes, entretanto cerca de quatro em cada dez profissionais da saúde nos Estados Unidos trabalham enquanto estão doentes.^(^^3^^)^ Esse estudo, realizado por meio de uma pesquisa *on-line* nacional, coletou dados de 1.914 profissionais de saúde durante a temporada de gripe no período entre 2014 e 2015. Os entrevistados relataram sintomas de gripe, definidos como a combinação de febre e tosse ou dor de garganta, e listaram os fatores que os levaram a não se ausentar do trabalho, de acordo com os principais resultados a seguir:

– Dos 1.914 profissionais de saúde pesquisados, 414 relataram sintomas de gripe. Destes, 183 (41,4%) relataram trabalhar por um período médio de 3 dias enquanto experimentavam esses sintomas.
– Os profissionais de saúde que desenvolvem suas atividades em hospitais tiveram a maior frequência de trabalho com sintomas de gripe (49,3%), em comparação com outros profissionais. Dentre eles, os mais propensos a trabalhar com gripe foram farmacêuticos (67,2%) e médicos (63,2%). Os enfermeiros apresentaram a frequência de 37,9% e outros profissionais de saúde de 32,1%.
– Os motivos mais comuns para os profissionais de saúde optarem em não se ausentar do trabalho incluíram o sentimento de que eles ainda poderiam realizar suas tarefas laborais, não apresentando esgotamento suficiente para permanecer em casa, a sensação de que não seriam veículos de transmissão para outras pessoas, o sentimento de dever profissional de estar presente no posto de trabalho para não comprometer e sobrecarregar os colegas e a dificuldade em encontrar um colega de trabalho para cobri-lo.

Em estudo anterior, ficou evidenciado que, em ambientes hospitalares, os pacientes internados expostos a pelo menos um profissional de saúde contaminado tiveram acima de cinco vezes mais chances de desenvolver gripe adquirida em hospital do que pacientes internados sem essa exposição.^(^^4^^)^

A saúde e o bem-estar dos pacientes estão em risco quando os profissionais de saúde contaminados optarem por não ficar em casa. Estratégias devem ser implementadas por instituições de saúde, incluindo a atualização das políticas de licença médica e educação para os profissionais de saúde realizarem escolhas saudáveis não apenas para si, mas também para seus colegas de trabalho e pacientes.^(^^3^^)^

Em tempos de pandemia pela doença pelo coronavírus 2019 (COVID-19), que pegou o mundo de surpresa e trouxe impacto na vida dos indivíduos em âmbito global, chamando a atenção pelo alcance que teve e pela velocidade com que se disseminou, surge a necessidade de atenção especial para a transmissão de doenças respiratórias entre profissionais de saúde e pacientes.^(^^5^^)^A transmissão de patógenos pelos profissionais de saúde representa um grande risco à saúde pública.^(^^1^^,^^3^^)^ Será que agora vamos direcionar nossos olhares para essa questão? Isso, aliás, no contexto atual, faz-se absolutamente urgente!

Encerramos este breve texto, mas o debate, cuja complexidade reconhecemos, deve continuar. A nosso ver, são pontos importantes para entender que, neste ano de 2020 do “avesso”, devemos priorizar essa discussão no meio acadêmico e assistencial, para garantir uma assistência segura. Fique em casa se estiver doente, profissional de saúde!
